# Targeting DNA Damage Response as a Strategy to Treat HPV Infections

**DOI:** 10.3390/ijms20215455

**Published:** 2019-11-01

**Authors:** N. Sanjib Banerjee, Dianne Moore, Cameron J. Parker, Thomas R. Broker, Louise T. Chow

**Affiliations:** Biochemistry and Molecular Genetics, University of Alabama at Birmingham, Birmingham, AL 35294, USA; salute@uab.edu (D.M.); cjparker@uab.edu (C.J.P.); broker@uab.edu (T.R.B.)

**Keywords:** HPV productive infection, cervical cancer, raft culture, DDR, Chk1 inhibitor, MK-8776

## Abstract

Mucosotropic human papillomaviruses (HPVs) cause prevalent anogenital infections, some of which can progress to cancers. It is imperative to identify efficacious drug candidates, as there are few therapeutic options. We have recapitulated a robust productive program of HPV-18 in organotypic raft cultures of primary human keratinocytes. The HPV E7 protein induces S phase reentry, along with DNA damage response (DDR) in differentiated cells to support viral DNA amplification. A number of small molecule inhibitors of DDR regulators are in clinical use or clinical trials to treat cancers. Here, we used our raft culture system to examine effects of inhibitors of ATR/Chk1 and ATM/Chk2 on HPV infection. The inhibitors impaired S-phase reentry and progression as well as HPV DNA amplification. The Chk1 inhibitor MK-8776 was most effective, reducing viral DNA amplification by 90–99% and caused DNA damage and apoptosis, preferentially in HPV infected cells. We found that this sensitivity was imparted by the E7 protein and report that MK-8776 also caused extensive cell death of cervical cancer cell lines. Furthermore, it sensitized the cells to cisplatin, commonly used to treat advanced cervical cancer. Based on these observations, the Chk1 inhibitors could be potential effective agents to be re-purposed to treat the spectrum of HPV infections in single or combination therapy.

## 1. Introduction

The prevalent human papillomaviruses (HPVs) infect mucosal or cutaneous epithelia. The mucosotropic HPVs can be sexually transmitted and are of major medical importance. The low-risk (LR) HPV-6 and -11 cause 90% of benign anogenital warts, whereas the high-risk (HR) HPVs such as types-16, -18 and closely related genotypes have oncogenic potential [1]. Most infections are cleared by host immune responses, whereas in a small fraction of patients, persistent infection by the HR types can lead to high grade intraepithelial lesions and cancers. Cervical cancers and derived cell lines are phenotypically negative for key tumor suppressor proteins p53 and the pRB family proteins because they continuously express the HR HPV E6 and E7 oncoproteins that destabilize these tumor suppressors [2]. Early screening and surgical removal of premalignant lesions have greatly reduced patient death in developed countries. However, in resource poor regions of the world, cervical cancer is a major cause of death. The highly efficacious prophylactic vaccines (Gardasil4 and Gardasil9) prevent infections by the targeted virus types [3]. However, due to the high costs and socio-economic factors, the take rates of the vaccines are highly variable within US and around the world. Since advanced HPV cancers are often recalcitrant to the standard chemo-radiation treatment, there is ongoing research and clinical trials in therapeutic and immunotherapeutic agents. However, there remains an urgent need to develop small molecule inhibitors that can effectively treat HPV premalignant lesions, thereby preventing virus production, transmission and lesion progression, especially in developing countries. The observation that HPV-16 is associated with about at least 30% of the head and neck cancers [4,5] further adds significance to the quest for effective therapeutic agents.

The amplification of the circular double-stranded HPV DNA genome of 7.9 kb and the assembly of the progeny virus requires a differentiating squamous epithelium [6]. Viral DNA replication depends on the viral E2 protein, which recognize the viral origin of replication and helps recruit the viral replicative helicase E1. The host supplies all remaining DNA replication enzymes, proteins and substrates. Organotypic raft cultures of primary human keratinocytes (PHKs) generate a stratified and differentiated squamous epithelium [7,8,9]. To support viral DNA amplification in differentiated keratinocytes, the viral oncoprotein E7 mediates the destabilization of p130, a pRB related protein, which maintains homeostasis of differentiated cells and suppress the expression of E2F-responsive replication genes. The loss of p130 leads to S-phase reentry essential to viral DNA amplification [10,11]. Utilizing the Cre-loxP recombination in early passages of PHKs, our lab has established a robust productive raft culture system, which supports high levels of HPV-18 DNA amplification and generates high titers of infectious HPV-18 virions [12]. HPV-18 E6 mutants unable to destabilize p53 do not support high levels of viral DNA amplification [12,13].

In normal mammalian cells, the DNA damage response (DDR) pathway is required to maintain replication fork stability and inhibit firing of dormant replication origins during S-phase [14,15,16,17]. The ATR-Chk1 pathway is activated by a stalled replication fork, which generates single-stranded DNA near newly replicated DNA, while ATM-Chk2 pathway is activated by collapsed replication forks and double-stranded DNA breaks [18,19]. The activation of both pathways during the S-phase ensures accurate chromosomal DNA duplication. Indeed, others and we have also shown that E7 or HPV infection induces a robust DNA damage response (DDR) in differentiated squamous cells that reenter the S-phase to support viral DNA amplification [12,20,21,22,23,24].

We showed that in PHK raft cultures productively infected with HPV-18, DDR target proteins Myt1 and Wee1 were phosphorylated and activated. In turn, Cdc25C was inactivated by phosphorylation on S216, unable to activate the cdc2/cyclin B1 complex, leading to their accumulation in the cytoplasm and a prolonged G2 phase. It is during this protracted G2 phase, the great majority of viral DNA amplification takes place [12,21]. Interestingly, p53 is stabilized by E7 expression [25,26,27]. Among many functions of the viral E6 protein [23,24], targeting p53 is critical, as elevated p53 due to defective E6 protein significantly hampers viral DNA amplification without triggering apoptosis [12,13].

HPV-31 genome amplification is also dependent on viral E7-dependent activation of ATM DNA damage response pathway in high calcium-promoted differentiation of submerged cultures of CIN612 cells derived from cervical dysplasia, or organotypic raft cultures of stably transfected and possibly immortalized human foreskin keratinocytes [24]. The authors reported E7 binding to ATM kinase triggered Chk2 activation and caspase-7 cleavage. HPV E7 also elevates MRN of the Mre11-Rad50-Nbs1 complex [20,24] and stabilizes Rad51 and BRCA1 [22], events instrumental to HPV-31 DNA amplification. Additionally, activated ATR-Chk1 pathway is required for efficient maintenance of HPV-16 genomic plasmids in the W12E keratinocytes in submerged cultures [23]. Several other DNA viruses also engage host DNA damage repair pathway, either to escape DDR induced replication block or facilitate virus replication [28,29].

In this study, we examined effects of inhibitors of the ATM/Chk2 and ATR/Chk1 pathways on productive HPV-18 DNA amplification in PHK raft cultures. In general, each inhibitor reduced cells in S-phase and cells in protracted G2 phases. Consequently, these compounds reduced viral DNA amplification. Among them, the Chk1 inhibitor MK-8776 was most effective and virtually abrogated HPV-18 DNA amplification because it also consistently induced DNA damage and apoptosis. We showed that the expression of E7 alone in PHK raft cultures conferred the sensitivity to the Chk1 inhibitor. Since cervical cancer cell lines continuously express HR HPV E6 and E7 oncogenes, we also examined the effects of MK-8776 on SiHa and CaSki cell lines. Our results showed that this Chk1 inhibitor induced extensive cell death when the cells were grown as submerged cultures or as raft cultures. Importantly, the combination of MK-8776 and cisplatin, a conventional therapeutic agent to treat advanced cervical cancer, is lethal to the cancer cell lines.

## 2. Results

### 2.1. Cytotoxic Effect of ChkI Inhibitor MK-8776 in HPV-18 Infected PHK Raft Cultures

**Figure 1:**
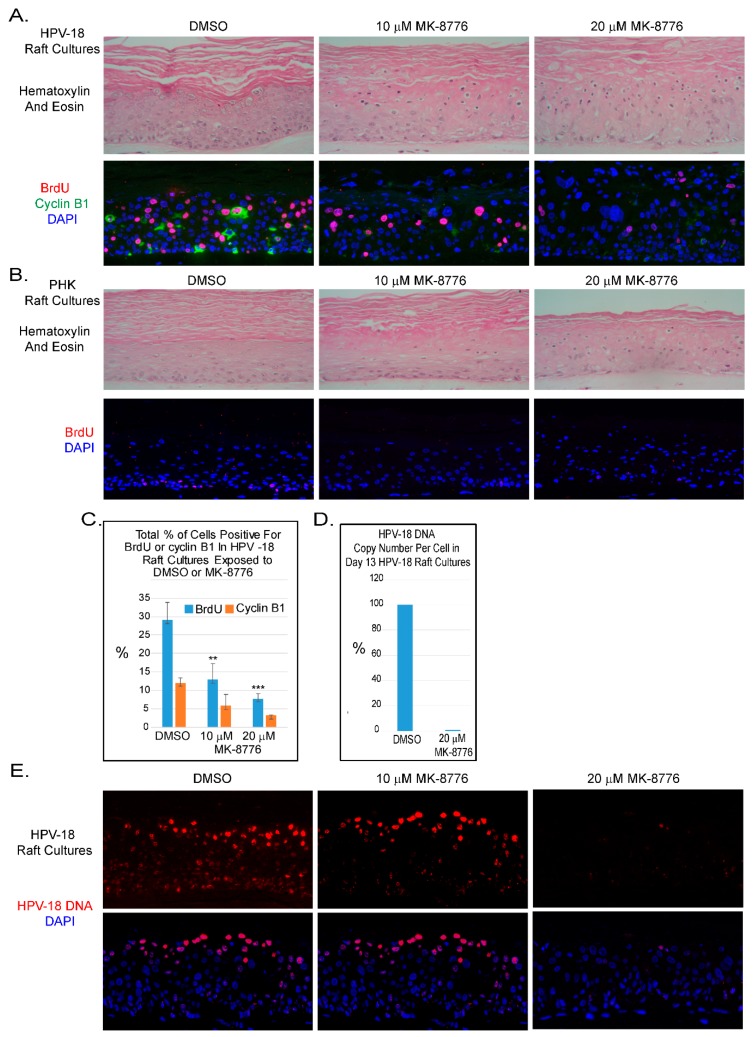
Chk1 inhibitor, MK-8776, reduces host DNA replication and abrogates productive amplification of human papillomavirus (HPV)-18 in raft cultures. (**A**) Four micron tissue sections of HPV-18 raft cultures exposed to DMSO or 10 and 20 µM MK-8776 from days 7 to 12. Upper row, hematoxylin and eosin staining, showing cytotoxic effects, evident from condensed nuclei in upper differentiated strata. Cells were enlarged at the higher MK-8776 treated cultures. Lower row, dual immunofluorescence (IF) assay to detect BrdU incorporation (red) and cytoplasmic cyclin B1 accumulation (green). (**B**) Four micron tissue sections of uninfected PHK raft cultures exposed to DMSO or 10 and 20 µM MK-8776 from days 7 to 12. Upper row, H&E staining. Cytotoxicity was much less pronounced than the HPV-18 raft cultures. Lower row, indirect immunofluorescence assay of BrdU incorporation (red) revealed host DNA replication in the basal layer was abrogated by MK-8776 exposures. (**C**) Bar-graphs of percentages of BrdU (blue) and Cyclin B1 (orange) positive cells in DMSO and MK-8776 treated (days 7–12) HPV-18 infected raft cultures. The data were collected from three non-overlapping microscopic fields from the same experiment. Statistical analyses were performed by using Microsoft excel to determine significance of difference (*p* values) indicated as ** (*p* < 0.05) or *** (*p* ≤ 0.005). (**D**) Bar-graph showing percentage of HPV-18 DNA copy number per cell in control and MK-8776 treated (days 7–13) raft cultures, as determined by quantitative real time PCR. (**E**) Upper row, detection of HPV-18 DNA amplification in raft culture tissue sections by FISH (fluorescent in situ hybridization). Bottom row, merged images with DAPI (blue)-stained nuclei. Nuclei were also detected with DAPI in A and B panels. Images were captured with 20× objective magnification.

The ATR-Chk1 pathway is a key regulator of replication checkpoint and DNA damage repair [30]. In recent years efficacy of small molecule inhibitors of Chk1 has been tested in combination with DNA damaging agents in clinical trials against solid tumors, myeloid leukemia and small cell lung carcinoma with some favorable outcomes [31,32,33,34]. We were particularly interested to examine the effects of MK-8776 because of its higher selectivity against Chk1 over Chk2 [35]. Based on the concentrations used in treating cancer cell lines [36,37,38] and the low toxicity in submerged PHKs (See Figure 7A), we exposed uninfected and HPV-18 infected PHK raft cultures to 10 or 20 µM MK-8776 for 6 to 8 days in parallel experiments. Histology of HPV-18 infected raft cultures revealed that exposure to 10 µM MK-8776 (days 6–12) induced cytotoxicity, characterized by cells with condensed nuclei suggestive of apoptosis, primarily in the upper strata. This toxic effect became wide spread at 20 µM (Figure 1A, upper row). At either concentration, MK-8776 had much reduced toxicity in uninfected raft cultures (Figure 1B, upper row). As reported previously [39], exposure to the DMSO vehicle had little effect in infected and uninfected cultures.

### 2.2. MK-8776 Effectively Inhibited Host DNA Replication and Cytoplasmic Cyclin B1 Protein Accumulation

Indirect Immunofluorescence (IF) assay showed that days 6–12 exposure of MK-8776 reduced S-phase cells as revealed by BrdU incorporation in the suprabasal strata of infected cultures (Figure 1A, lower row). BrdU-positive suprabasal cells enumerated from three fields from one experiment were reduced to 13 and 8 percent of the total DAPI-positive nuclei in the presence of 10 and 20 µM MK-8776, respectively, whereas 29 percent of the suprabasal DAPI-positive nuclei were positive for BrdU in the DMSO-treated control (Figure 1C). Similarly, percentage of cells with cytoplasmic cyclin B1 relative to total number of DAPI-positive nuclei was reduced from 12% to about 6 and 3 percent (Figure 1A,C). Similar trend in reduction of S-phase and cytoplasmic cyclin B1-positive cells were observed in two other independent experiments. These results indicate that the S-phase cells and their progression to G2 were reduced. We also noted that BrdU incorporation in the basal cells of uninfected tissue was abrogated (Figure 1B lower row).

### 2.3. MK-8776 Effectively Inhibited viral DNA Amplification

We then analyzed HPV-18 DNA amplification. In cultures treated with vehicle, Fluorescent In-Situ Hybridization (FISH) revealed that high signals were observed predominantly in the upper differentiated strata while low signals were detected in lower spinous layers (Figure 1E). Six days of exposure to 10 µM MK-8776 (days 7–13) significantly reduced signals in the lower strata. However, at 20 µM, HPV-18 DNA was barely detectable (Figure 1E). Residual signals were primarily observed in small HPV-18 foci in some cells. Similar data were obtained from two additional independent experiments. Quantitative PCR analysis from one of the experiments confirmed that HPV-18 DNA copy number per cells in the culture treated with 20 µM MK-8776 were reduced to less than 1% of the control (Figure 1D).

### 2.4. Mechanism of MK-8776 induced Inhibition of HPV-18 DNA Amplification

**Figure 2:**
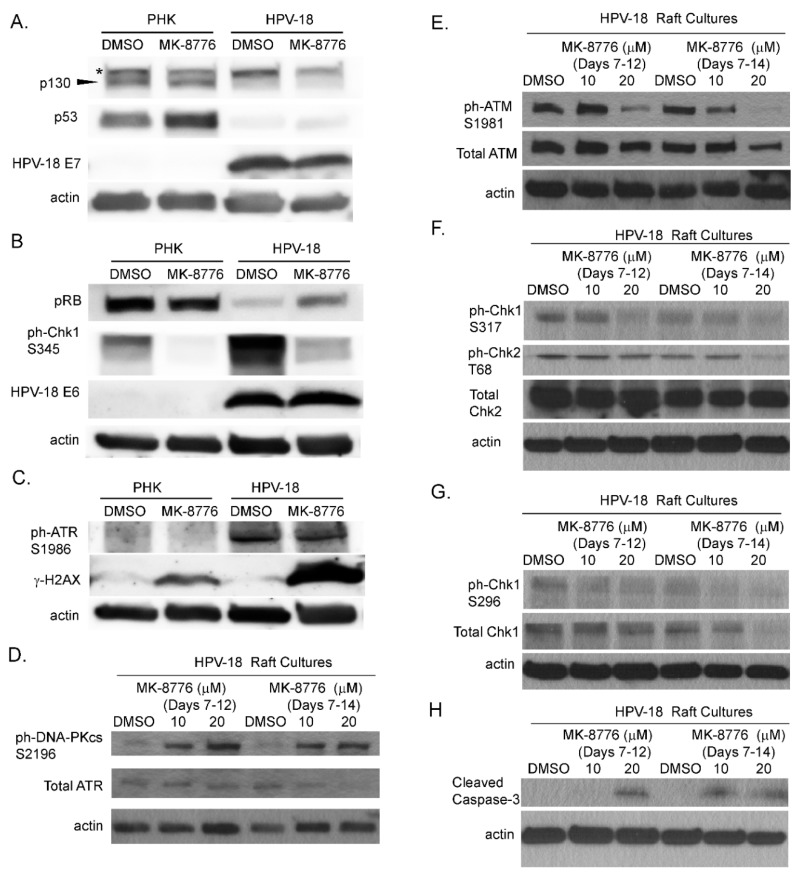
Effects of MK-8776 exposure on HPV-18 E6 and E7 and DNA damage response (DDR) proteins, as well as a biomarkers of DNA damage and apoptosis in raft culture lysates. (**A**–**C**) Uninfected (PHK) and infected (HPV-18) raft cultures were exposed to DMSO or 20 µM MK-8776 from days 7 to 12 and harvested on day 12. Lysates were probed by immunoblot assays to detect HPV-18 E6, E7, pRB, p130, p53, ph-Chk1 S345, ph-ATR S1986 and γ-H2AX S139. (**D**–**H**) Immunoblot analysis of lysates from HPV-18 raft cultures exposed to DMSO, 10 or 20 µM MK-8776 from days 7–12 or days 7–14 and harvested on days 12 or 14, respectively. Immunoblots were performed to detect total ATR, total ATM, ph-ATM S1981, total Chk2, ph-Chk2 T68, ph-Chk1 S317, total Chk1, ph-Chk1 S296 and cleaved caspase. Actin served as loading control for each set of immunoblots. Arrowhead points to p130 protein band. * denotes a non-specific protein detected by the p130 antibody.

Next, we analyzed the expression and levels of viral and host proteins in raft cultures to explore the underlying mechanism of the inhibitory effects of MK-8776 on virus DNA amplification. Steady state levels of HPV E6, E7, their primary target proteins, p53, pRB and p130 and virus induced DDR host proteins from the raft culture lysates were analyzed by immunoblot analyses. Upon exposure from days 7–12, 20 µM MK-8776 neither reduced steady state levels of HPV-18 E6 and E7 proteins, nor increased their target host proteins, (Figure 2A,B). Thus, the expression and functions of the E6 and E7 proteins are independent of the Chk1 function. As reported previously, ph-ATR and ph-Chk1 were elevated by HPV infection relative to uninfected PHK raft cultures (Figure 2B,C). Upon exposure to the inhibitor (days 7–12), there was little or no change in total or phosphorylated ATR, but ph-Chk1 (S345) was significantly reduced (Figure 2B,C,F), verifying the inactivation of Chk1. Upon a prolonged exposure to 20 µM MK-8776 (days 7–14), total ATR, total Chk1 and ph-Chk1 (S296) in the infected raft cultures were all dramatically reduced (Figure 2D,G). Interestingly, in PHK raft cultures, ph-Chk1 (S345) was reduced to undetectable levels. We reason that Chk1 inhibition abrogated S-phase in the uninfected raft cultures (Figure 1B), thereby abolishing ATR-mediated ph-Chk1. We note that the reduction of ph-Chk1 by MK-8776 in our uninfected PHK raft cultures is in contrast to earlier reports where, Chk1 inhibition was associated with increased ph-ATR and ph-Chk1 (S317, S345) [40,41]. However, these latter studies were based on different experimental systems, in which transformed cell lines were subjected to external genotoxic stress, such as ionizing radiation or exposure to hydroxyurea or gemcitabine.

Since the DDR/Chk1 pathway regulates replication fork fidelity, Chk1 inhibition would induce accumulation of DNA damage leading to replication stress and abortive DNA replication. Phosphorylation of H2AX (γ-H2AX) is a signature of DNA damage. Elevated γ-H2AX protein was indeed detected by immunoblot in HPV-18 raft cultures following the exposure to 20 µM MK-8776 from days 7 to 12 (Figure 2C). In contrast, the treated uninfected PHK raft cultures exhibited only very weak signals (Figure 2C), as might be expected from the observation that the inhibitor abrogated DNA replication in basal cells. Untreated cultures exhibited little or no signals.

**Figure 3:**
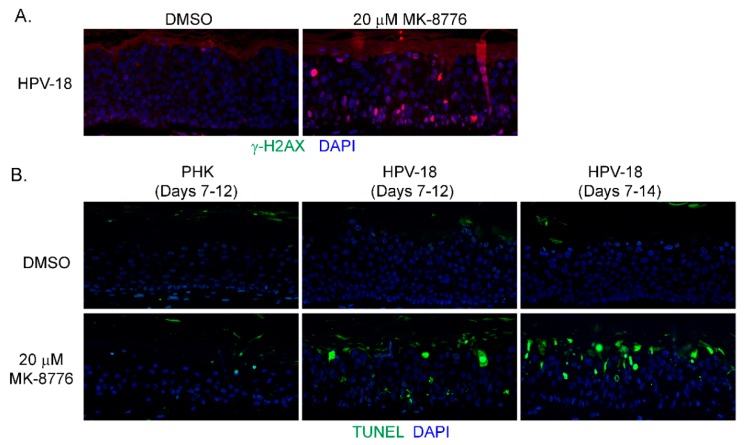
MK-8776 induced DNA damage and apoptosis in HPV-18 infected or uninfected PHK raft cultures. (**A**) Indirect immunofluorescence assay to detect γ-H2AX in HPV-18 raft cultures exposed to 20 µM MK-8776 (days 7–14) but not in DMSO treated control. (**B**) TUNEL assay to detect apoptotic nuclei (green signals) in PHK or HPV-18 raft cultures exposed to DMSO or 20 µM MK-8776 for days 7–12 or 7–14. Nuclei in both A and B were detected with DAPI (blue). Images were captured with 20× objective.

In mammalian cells, γ-H2AX is induced by ATM as a result of double-stranded DNA break (DSB) [42], by activated ATR when DNA suffers single-stranded DNA breaks due to stalled replication fork [17], or by DNA-PKcs during DNA fragmentation in apoptotic cells [43]. Therefore, we probed for ATM, and DNA-PKcs proteins in immunoblots of lysates of raft cultures exposed to 20 µM MK-8776 from days 7 to 12 or 14. Phosphorylated ATM and Chk2 both decreased after the 8-day exposure to MK-8776 (Figure 2E,F). In contrast, phosphorylated DNA-PKcs protein was elevated after exposure to 6 or 8 days (Figure 2D). At 10 mM MK-8776, the compound did not induce cleaved caspase 3 (Figure 2H, days 7–12 treatment), nor γ-H2AX (data not shown), but both host and viral DNA replication was reduced relative to the control (Figure 1C,E). Thus, we interpret that Chk1 inhibition impeded host and viral DNA replication by preventing the resolution of stalled replication forks, events that preceded accumulation of DNA damage and apoptosis. The immunoblots also showed that 20 µM MK-8776 treated (6 or 8 days) HPV-18 infected raft cultures had elevated cleaved caspase 3 (Figure 2H), whereas no signals were detected in untreated HPV-18 raft cultures. Thus, we suggest that elevated ph-DNA-PKcs were responsible for the γ-H2AX induction prior to cells undergoing apoptosis. In accordance, IF probing of tissue sections revealed stochastic accumulation of γ-H2AX only in the nuclei of MK-8776 treated HPV-18 raft cultures (Figure 3A). To verify apoptosis, we performed a TUNEL assay. Upon exposure to 20 µM MK-8776 from days 7–12, a small number of TUNEL positive cells were detected in the HPV-18 raft cultures. However, the number of such cells increased significantly after a prolonged exposure from days 7–14 (Figure 3B). In contrast, TUNEL-positive spinous cells were rarely detected in the untreated HPV-18 cultures (Figure 3B).

These results are at variance with the induction of γ-H2AX and cleaved caspase-7 in raft cultures of HPV-31 infected human foreskin keratinocytes (HFK) upon extensive passage [24]. In our raft cultures of early passage HPV-18 infected PHK raft cultures, we did not detect cleaved caspase-3, nor elevated levels of γ-H2AX relative to untreated, uninfected or HPV-18 infected raft cultures using IF or prolonged exposure of immunoblots ([12,13,39,44,45] and our unpublished observation).

**Figure 4:**
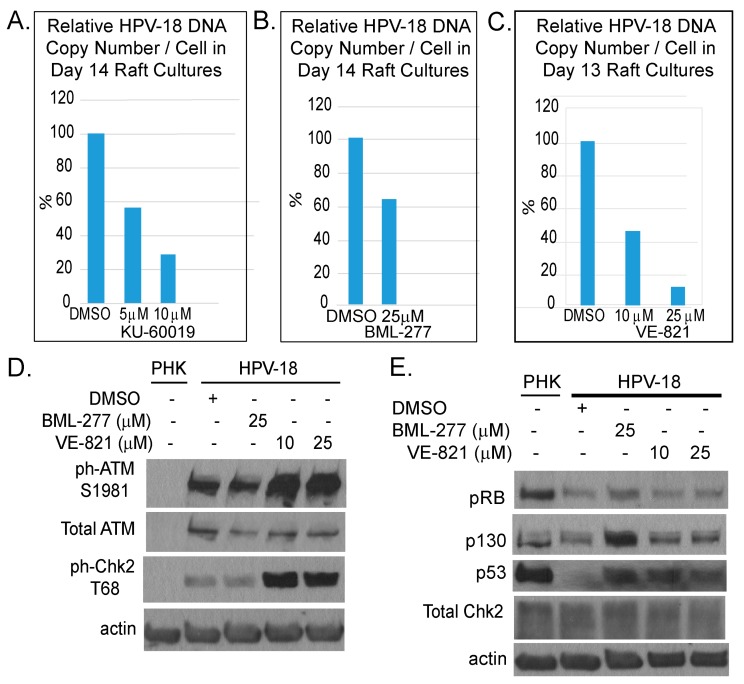
Comparison of inhibitory effects of inhibitors of ATM (KU-60019), Chk2 (BML-277) and ATR (VE-821). (**A**–**C**) Bar chart depicts relative HPV-18 DNA copy number per cell as determined by real time qPCR of DNA from raft cultures exposed to KU-60019 (days 7–14), BML277 (days 7–14) or VE-821 (days 6–13) and harvested on last days of treatment. (**D**,**E**) Immunoblot analyses of lysates from untreated PHK raft cultures and from DMSO-, BML-277 or VE-821 treated HPV-18 raft cultures. (**D**) HPV-18 infection activates DDR response relative to uninfected cultures. VE-821-treated HPV-18 raft cultures had elevated ph-ATM S1981 and ph-Chk2 relative to DMSO-treated cultures. (**E**) Treated HPV-18 infected cultures had increased p130 and p53 relative to untreated cultures; but, treatments did not alter total Chk2 protein level. Actin served as loading control in both gels.

We also examined the effects of inhibitors of ATR (VE-821), ATM (KU-60019) and Chk2 (BML-277). Each was investigated in two independent experiments. In general, the inhibitors reduced S-phase cells in the suprabasal strata of HPV-18 infected raft cultures (data not shown) and suppressed viral DNA amplification (Figure 4A–C). However, they were less effective than the Chk1 inhibitor (compare to Figure 1D). We note that, in contrast to Chk1 inhibitor, ATR inhibitor elevated total and ph-ATM as well as ph-Chk2 (Figure 4D). Additionally, p130 and p53 protein levels were elevated relative to DMSO treated cultures (Figure 4E), whereas there was no change in these host proteins upon exposure to the Chk1 inhibitor (Figure 2). This differential effect between the Chk1 and ATR inhibitors on the ATM/Chk2 pathway might have contributed to the different efficacies in inhibiting viral DNA amplification.

### 2.5. HPV-18 E7 Promoted S-phase re-entry Sensitizes Host Cells to MK-8776 induced DNA Damage and Apoptosis

**Figure 5:**
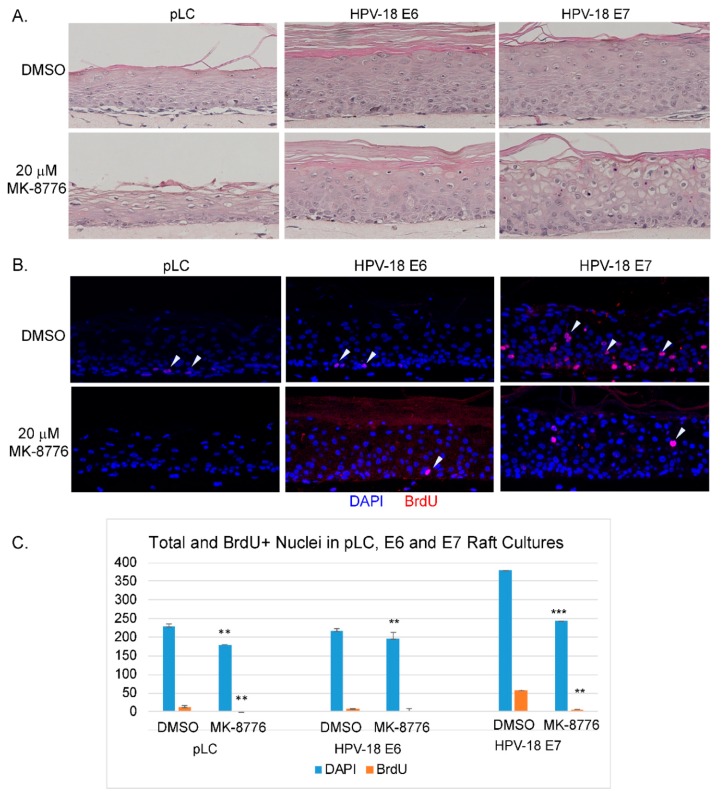
HPV-18 E7 sensitizes PHK raft cultures to Chk1 inhibitor MK-8776. (**A**) Hematoxylin and eosin staining of raft cultures PHK containing empty vector (pLC), HPV-18 E6 or HPV-18 E7. The cultures were exposed to DMSO or 20 µM MK-8776 from days 8 to 12. Enlarged vacuolated cells with condensed nuclei were most prominent in the E7-expressing culture. (**B**) IF assay to detect BrdU (red) incorporation in the above raft cultures. Arrow heads indicate to the BrdU signals. (**C**) Significant inhibition of DNA replication by MK-8776 in raft cultures. Quantitative representation of total and BrdU positive nuclei from 3 microscopic fields in raft culture sections. The *p* values (determined by a student’s *t*-test using MS Excel) are indicated with ** (<0.05) or *** (<0.005). Images were captured with 20× objective.

**Figure 6:**
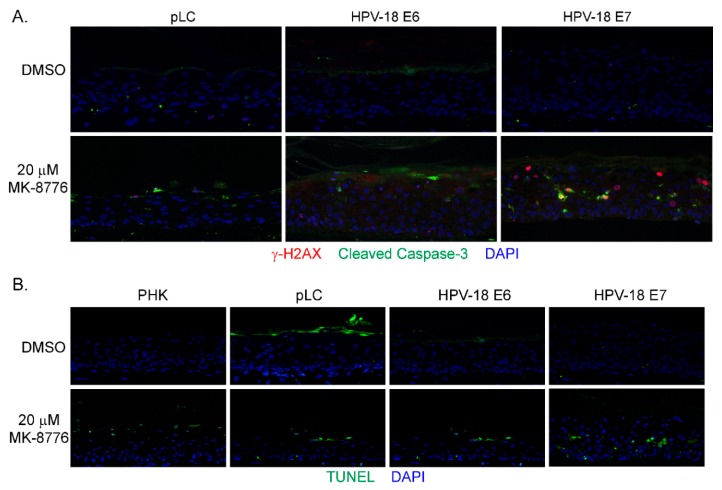
HPV-18 E7 sensitizes PHK raft cultures to Chk1 inhibitor MK-8776. (**A**) Detection of biomarkers of DNA damage (γ-H2AX, red) and apoptosis (cleaved caspase 3, green) by dual IF in raft culture sections. (**B**) Apoptosis was detected by TUNEL assay on sections from raft cultures of uninfected PHK (PHK), PHK containing empty vector (pLC), HPV-18 E6 or E7. Images were captured with 20× objective. Only E7-expressing culture exhibited signals in the spinous strata. The Most signals in the other three cultures were observed in stratum corneum.

Since the toxic effects of the Chk1 inhibitor on the HPV-18 infected raft cultures is preferentially observed in the differentiated cells that reentered the S-phase, we surmised that the expression of E7 or E6 and E7 is responsible for sensitizing the cells. To test this hypothesis, raft cultures of PHKs infected with retroviruses expressing HPV-18 E6, E7 or the empty vector were prepared. Consistent with our previous observations [46], nuclei of suprabasal cells positive for BrdU were only observed in the E7-transduced raft cultures, indicative of E7-induced host DNA replication (Figure 5B). In contrast, BrdU incorporation was detected only in the basal cells in the vector- or E6-transduced raft cultures. Exposure to 20 µM MK-8776 (days 8 to 12) significantly reduced total and BrdU-positive nuclei in all three raft cultures (Figure 5B,C). By histology, MK-8776 exposure induced vacuolated large cells with condensed nuclei, were most prominent in the HPV-18 E7-transduced raft culture (Figure 5A). Dual IF assays revealed many cells in the differentiated strata of the E7 raft cultures had nuclear γ-H2AX, often colocalizing with cleaved caspase-3 in the cytoplasm (Figure 6A). Some areas in HPV-18 E7 raft cultures were devoid of nuclei, suggestive of apoptosis (Figure 6A). Very few cells in HPV-18 E6- or vector-transduced raft cultures had nuclear γ-H2AX or cleaved caspase-3 signal. Indeed, TUNEL assay detected apoptosis within the differentiated strata of E7 raft cultures. In contrast, sparse TUNEL signal could be detected in superficial stratum corneum of the uninfected PHK, pLC or E6 raft cultures. Thus, E7 induced S-phase reentry in the living spinous cells made them sensitive to MK-8776 induced DNA damage and apoptosis. Since E7 expression in PHK raft culture stabilizes p53, which is degraded when E6 is coexpressed [27], we concluded that the destabilization of p53 by E6 plays little or no role in Chk1 inhibitor-induced cell death.

### 2.6. MK-8776 Sensitizes Cervical Cancer Cell Lines to Cisplatin

**Figure 7:**
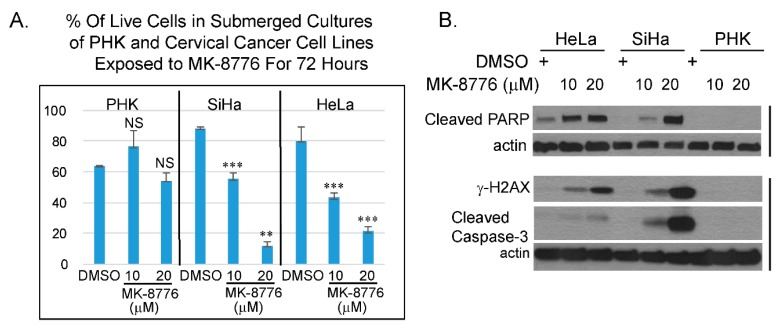
Cervical cancer cell lines are more sensitive to MK-8776 relative to uninfected PHK in submerged cultures. (**A**) A bar-graphs showing percentage of live cells of PHKs, cervical cancer cell lines, SiHa or HeLa, as determined by trypan blue assay. Data were averages from triplicate. *** indicates statistically significant difference (*p* < 0.005) of living cells in treated cultures relative to DMSO treated controls. (**B**) Immunoblot analyses of lysates from submerged cultures of HeLa, SiHa and PHK revealed that MK-8776 induced cleaved PARP, γ-H2AX, and cleaved caspase 3 in concentration dependent manner in HeLa and SiHa, but not in PHKs. Actin served as loading control.

In combination with DNA damaging agent, Chk1 inhibitor, MK-8776 was tested in clinical trials against some solid tumors, lymphoma and myeloid leukemia [31,32]. MK-8776 sensitizes HeLa cells to X-ray irradiation [47]. HeLa cells are derived from an HPV-18 containing cervical cancer. Whether this inhibitor has a direct effect on cervical cancer cell lines has not been investigated. Thus, we examined if MK-8776 is toxic to HPV cervical cancer cell lines HeLa and SiHa. Trypan blue assays were performed on submerged cultures of PHK, HeLa and SiHa. The results showed that the toxicity in HeLa and SiHa upon 72 h exposure to MK-8776 was concentration dependent. At 20 µM, there was significant toxicity, whereas PHKs were less sensitive (Figure 7A). Immunoblot analyses also confirmed that MK-8776 induced the accumulation of γ-H2AX, cleaved PARP and cleaved caspase-3 in submerged cultures of HeLa and SiHa, but not in PHKs (Figure 7B). We reason that the relatively low sensitivity in PHKs might be attributed to their intact cell cycle surveillance mechanisms mediated by the tumor suppressor proteins p53, pRb and but it is also possible that the cervical cancer cell lines have additional mutations that further compromised their cell cycle control.

**Figure 8:**
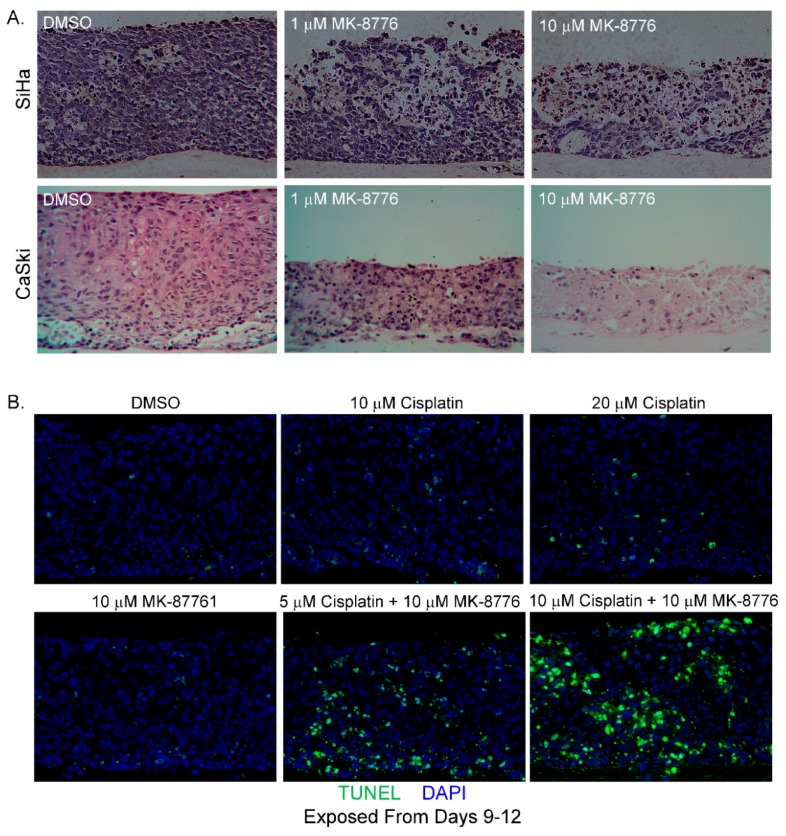
MK-8776 is cytotoxic to raft cultures of cervical cancer cell lines and increases their sensitivity to cisplatin. (**A**) Hematoxylin and eosin staining of tissue sections from SiHa and CaSki raft cultures, exposed to DMSO or MK-8776 at indicated concentrations from days 7 to 13 (SiHa) or 6–12 (CaSki). Cultures were harvested on last days of treatment. Cytotoxicity is evident from the degenerated tissues. (**B**) TUNEL assay showing apoptotic nuclei (green) in raft cultures of CaSki exposed to MK-8776, cisplatin, or both at indicated concentrations from days 9 to 12. Images were captured with 20× objective.

We then evaluated the effect of MK-8776 in raft cultures of SiHa cells on days 7–13 and CaSki cells, another HPV-16 positive cervical cancer cell line, on days 6–12. The cultures were harvested on last day of treatment. With increasing concentrations, MK-8776 exposure induced extensive cytotoxic effects in both raft cultures (Figure 8A). In our experience, raft cultures of CaSki cells were always more robust and less sensitive to various inhibitors than raft cultures of SiHa cells. Indeed, this was the case here. Higher toxicity was observed at 1 µM concentration of MK-8776 in SiHa than in CaSki raft cultures. Being derived from an invasive adenocarcinoma, HeLa does not stratify in raft cultures.

**Figure 9:**
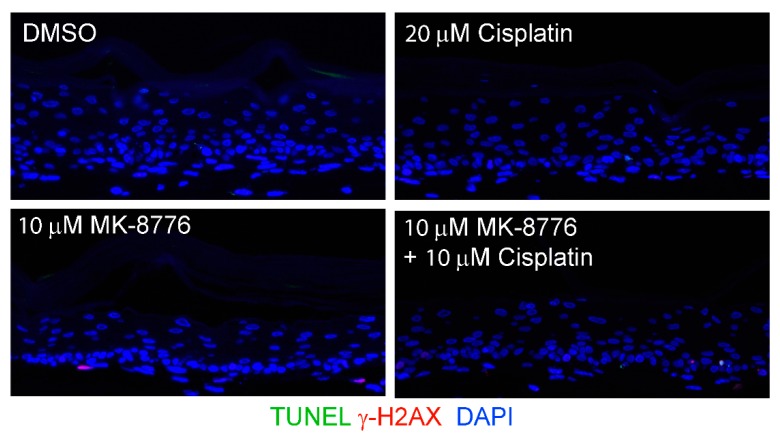
Combination of cisplatin and MK-8776 is weakly toxic on uninfected PHK raft cultures. TUNEL assay and γ-H2AX was probed in raft cultures of uninfected PHKs exposed to DMSO, 20 µM cisplatin, 10 µM MK-8776 and combination of cisplatin and MK-8776, both at 10 µM, from days 9 to 12 before harvest. Images were captured with 20× objective. Note that the epithelium was thinner in all three treatments attributed to a blockade of basal cell proliferation. However, there is hardly any γ-H2AX induction or TUNEL signals.

We then examined CaSki cell killing by MK-8776 in combination with cisplatin (Figure 7 and Figure 8B), a chemotherapeutic drug used in the standard of care to treat advanced cervical cancer. When added alone, treatment of CaSki raft cultures for 3 days (days 9–12) with MK-8776 at 10 M or cisplatin at 10 or 20 µM did not induce significant apoptosis (Figure 8B). Parallel raft cultures were also exposed to both agents. The presence of 10 µM MK-8776 sensitized the cultures to 5 M of cisplatin, exhibiting higher TUNEL signals than 10 M of either agent alone. Upon simultaneous treatment with 10 µM cisplatin and 10 µM MK-8776 for 3 days, highly effective cell killing led to wide spread TUNEL-positive signals. In contrast, very little signal of DNA damage or apoptosis was detected in PHK raft cultures exposed (days 9–12) to cisplatin (20 µM), MK-8776 (10 µM) or both (10 µM each; Figure 9). Thus, Chk1 inhibition and cisplatin are synthetic lethal against cervical cancer cell lines when cell cycle progression was not curbed before extensive DNA damages were repaired. In contrast, in PHK raft cultures, DNA replication was halted by an intact cell cycle surveillance, minimizing DNA damage and apoptosis.

## 3. Discussion

HPV E7 induces S-phase reentry in the post-mitotic differentiated epithelial strata. It also induces ATM and ATR, as well as other DDR proteins, causing prolonged G2 and enabling viral DNA amplification. Thus, DDR proteins play a direct role during HPV DNA amplification [21,22,23,24,44]. In this study, we tested our hypothesis that inhibitors of the DDR pathways would inhibit viral DNA amplification. In productively infected HPV-18 raft cultures, inhibitors of ATM, Chk2, ATR and Chk1, each reduced BrdU positive nuclei and cells with cytoplasmic cyclin B1 accumulation in the differentiated strata, indicative of reduced S-phase reentry and progression to the G2 phase. Since HPV DNA amplifies primarily in the protracted G2 phase, each of the inhibitor reduces viral DNA amplification. Amongst the inhibitors tested, the Chk1 inhibitor MK-8776 is most effective. It reduces S-phase cells by about 27.5% and the viral copy number per cells by 99%, relative to the untreated control cultures (Figure 1). We note that the inhibition of viral DNA amplification is more severe than the reduction of suprabasal BrdU-positive cells. We suggest that stalled or damaged replicating HPV DNA is preferentially degraded relative to non-replicating viral DNA. This interpretation is consistent with the detection of very small HPV DNA foci in inhibitor-treated raft cultures observable at higher magnification (data not shown). This non-replicating viral DNA pool would then account for the continued expression of viral E6 and E7 proteins (Figure 2). In addition to a direct effect on viral DNA amplification, prolonged exposure to MK-8776 induced host DNA damage and apoptosis in some of the differentiated cells, contributing to further reduction of total viral DNA copy numbers in the treated tissues. Our studies also show that E7 alone confers the sensitivity of HPV-infected raft cultures to the cytotoxic effects of MK-8776 in the differentiated strata in which E7 promotes S phase reentry (Figure 5B,C).

Our observations in the current study highlight the importance of DDR proteins, particularly Chk1, in the context of HPV infection. Intriguingly, the ATR inhibitor VE-821, though inhibited Chk1 activation, it elevated phosphorylated ATM and Chk2 (Figure 2), possibly by compensatory mechanisms. The higher efficiency in reducing viral DNA by the Chk1 inhibitor MK-8776 relative to ATR inhibitor VE-821 might be explained by the additional ability of MK-8776 to reduce phosphorylated ATM and chk2, both of which also play a role in support viral DNA amplification [21,24]. The observation that the elevated p-ATM/p-Chk2 induced by the ATR inhibitor did not rescue the inhibitory effect on the ATR/Chk1 functions also imply and the activated ATR/Chk1 and ATM/Chk2 have distinct roles in HPV DNA amplification, as previously proposed in SV40 replication [48].

We further show that cervical cancer cells caused by the HR HPV-18 and HPV-16 are more sensitive to the Chk1 inhibitor MK-8776 than productively infected PHK raft cultures. MK-8776 and cisplatin, the standard chemotherapeutic agent to treat metastatic cervical cancer, are synthetic lethal to these cells. The elevated sensitivity of cervical cancer cell lines may be attributed to high level of the viral oncoprotein expressions or some additional defects in DNA damage repair pathways in these cancer cells. The fact that cervical cancers are highly deficient in the two major tumor suppressor proteins, p53 and pRB family, that regulate cell cycle control, may make them more susceptible to Chk1 inhibitor than other solid tumors caused by dysregulation of other signaling pathways.

In summary, our data suggest that ATR/Chk1 signaling is a critical component not only for the cellular DNA replication, it also has a major impact on viral DNA amplification and the viability of cervical cancer cell lines. Further studies will be needed to determine whether Chk1 inhibitor is a candidate agent in combination therapies to improve treatment of advanced HPV cancers.

## 4. Materials and Methods

### 4.1. Cells and Organotypic Raft Culture

Primary human keratinocytes were isolated from neonatal foreskin discarded from elective circumcision surgery under UAB approved IRB protocol. Only early passages (P0 or P1) were used. A Cre-recombinase encoding plasmid, pCAGGS-nlsCre [49] and a recombinant plasmid pNeo-LOXP-HPV-18, in which the HPV-18 genome was flanked by LoxP sites, were transfected into PHKs to generate HPV plasmids via Cre-mediated recombination [12]. Transfected PHKs were selected with G418 for 2 days and expanded for 2–3 days without passaging and seeded on dermal equivalent (DE) comprising collagen and mouse J2 3T3 fibroblasts. The assembly was raised to air media interphase (day 0) and cultured in 5% CO2 incubator. Inhibitors or the vehicle DMSO were added to the culture medium as indicated for each experiment. The medium was refreshed every other day and the cultures were harvested on days specified for each experiment. BrdU (100 µg/mL) was added to the media during the final 6 h to mark S-phase cells. One raft culture of each of the vehicle-treated control or inhibitor treated arm was formalin fixed and paraffin embedded (FFPE) for in situ analysis. Additional parallel cultures were frozen in liquid nitrogen and processed for biochemical analyses. Experiments were conducted 2–3 times with different batches of PHKs.

Raft cultures of PHKs transduced with retroviruses containing the HPV-18 URR-driven HPV-18 E6, HPV-18 E7 or the empty vector (pLC) were prepared as described [46,50] and exposed to Chk1 inhibitor from days 8 to 12 and then processed into FFPE on day 12. Cervical cancer cell lines, CaSki, SiHa and HeLa, were cultured in DMEM supplemented with 10% FBS. CaSki and SiHa were also grown as raft cultures as just described. The cultures were treated with MK-8776 from days 6–12 (for CaSki) or days 7–13 (for SiHa), and harvested on the last day of treatment. For combination drug study, the CaSki raft cultures were treated on days 9–12 with MK-8776, the Chk 1 inhibitor, cisplatin or both, harvested on day 12 and processed by FFPE.

### 4.2. Inhibitors

MK-8776 (chk1 inhibitor) and BML-277 (chk2 inhibitor) were purchased from ApexBio (Houston, TX, USA). KU-60019 (ATM inhibitor) was sourced from LC-Laboratories (Woburn, MA, USA), and VE-821 (ATR inhibitor) was bought from Sellekchem (Houston, TX, USA). Inhibitors were dissolved in DMSO per the manufacturer’s direction to prepare 1000× concentrated stocks. Inhibitor concentrations were selected based on published reports in various cancer cells lines (that do not contain HPVs) and on their low toxicity in submerged PHK cultures (Figure 7A, and data not shown).

### 4.3. Histology

Four µm sections of FFPE raft cultures of uninfected or productively infected with HPV-18 and of cervical cancer cell lines, Caski or SiHa cells were deparaffinized. Tissue histology was revealed by standard hematoxylin and eosin staining. Images were captured under Olympus BX-63 microscope fitted with DP-73 camera using cellSens software. Image processing to construct composite images was conducted with Adobe Photoshop and Illustrator.

### 4.4. In Situ Molecular Assays of Raft Cultures

Effect of ATM and Chk1 inhibitors on HPV-18 infected and uninfected PHK raft cultures were performed in three independent experiments. Raft cultures exposed to VE-821 or BML-277 were assessed in two independent experiments. Experiments conducted with cervical cancer cells presented in Figure 8 were done once. Indirect immunofluorescence detection of host proteins was performed as described [42]. Fluorescent DNA in situ hybridization (DNA-FISH) to detect viral DNA amplification was performed as described [12]. The following antibodies were used to probe host targets: mouse anti-BrdU (ZBU30, Thermofisher, Waltham, MA, USA), anti-Cyclin B1 (ab181593, Abcam, Cambridge, MA, USA), Gamma γ-H2AX (ab22551, Abcam,) and cleaved caspase 3 (Cat. 9661, Cell Signaling Tech, Danvers, MA, USA). TUNEL was performed using an in situ Cell Death Detection Kit, Fluorescein (Roche-11684795910, from Millipore Sigma, St. Louis, MO, USA). UV filter set from Chroma was used to visualize in red, green and blue fluorescence channels and images were captured as described above. The same image capture setting was used for all the specimens within each experiment. Image processing was carried out with Adobe Photoshop and Illustrator. For enumeration, DAPI-positive nuclei, BrdU positive nuclei as well as cytoplasmic cyclin B1 positive cells were counted from three independent microscopic fields from one experiment with count and measure application of cellSens. Standard deviation and *t*-test was performed with Microsoft Excel to determine standard error and *p* values.

### 4.5. Immunoblot

Frozen PHK and HPV-18 infected raft cultures were homogenized in mammalian cell lysis buffer and protein quantified as described [19]. Fifty or 100 µg of total protein were resolved in 5–15% gradient SDS-PAGE and wet transferred to PVDF membranes. Immunoblots were blocked in 5% nonfat dry milk or 5% BSA, probed with antibodies as described by the antibody suppliers. Signals were detected by GE Healthcare Amersham ECL Western Blotting Detection Reagents (Fisher Scientific, Waltham, MA, USA). The sources of various antibodies are as follows: HPV-18 E6, HPV-18 E7, (Santa Cruz (Dallas, TX, USA); p53 (DO7, Leica Biosystems-Novacastra Buffalo Grove, IL, USA); pRB, p130 (BD Biosciences. San Jose, CA, USA); total ATM, phospho-ATM (S1981; Abcam; Cambridge, MA, USA); ATR, total Chk1, ph-Chk1 (S296), ph-Chk1 (S345), Chk2, pChk2 (T68), γ-H2AX (S139) and cleaved PARP and cleaved caspase 3 (Cell Signaling Technologies; Danvers, MA, USA). Each experiment was repeated two or more times.

## References

[1] Hausen H.Z. (2009). Papillomaviruses in the causation of human cancers—A brief historical account. Virology.

[2] DeFilippis R.A., Goodwin E.C., Wu L., DiMaio D. (2003). Endogenous Human Papillomavirus E6 and E7 Proteins Differentially Regulate Proliferation, Senescence, and Apoptosis in HeLa Cervical Carcinoma Cells. J. Virol..

[3] Schiller J., Lowy D. (2018). Explanations for the high potency of HPV prophylactic vaccines. Vaccine.

[4] De Martel C., Plummer M., Vignat J., Franceschi S. (2017). Worldwide burden of cancer attributable to HPV by site, country and HPV type. Int. J. Cancer.

[5] Ndiaye C., Mena M., Alemany L., Arbyn M., Castellsagué X., Laporte L., Bosch F.X., De Sanjose S., Trottier H. (2014). HPV DNA, E6/E7 mRNA, and p16INK4a detection in head and neck cancers: A systematic review and meta-analysis. Lancet Oncol..

[6] Chow L.T., Broker T.R. (2013). Human Papillomavirus Infections: Warts or Cancer?. Cold Spring Harb. Perspect. Boil..

[7] Dollard S.C., Demeter L.M., Reichman R.C., Broker T.R., Chow L.T., Wilson J.L., Bonnez W. (1992). Production of human papillomavirus and modulation of the infectious program in epithelial raft cultures. OFF. Genes Dev..

[8] Doorbar J. (2016). Model systems of human papillomavirus-associated disease. J. Pathol..

[9] Lee D., Norby K., Hayes M., Chiu Y.-F., Sugden B., Lambert P.F. (2016). Using Organotypic Epithelial Tissue Culture to Study the Human Papillomavirus Life Cycle. Curr. Protoc. Microbiol..

[10] Genovese N.J., Banerjee N.S., Broker T.R., Chow L.T. (2008). Casein Kinase II Motif-Dependent Phosphorylation of Human Papillomavirus E7 Protein Promotes p130 Degradation and S-Phase Induction in Differentiated Human Keratinocytes▿. J. Virol..

[11] Genovese N.J., Broker T.R., Chow L.T. (2011). Nonconserved Lysine Residues Attenuate the Biological Function of the Low-Risk Human Papillomavirus E7 Protein▿. J. Virol..

[12] Wang H.-K., Duffy A.A., Broker T.R., Chow L.T. (2009). Robust production and passaging of infectious HPV in squamous epithelium of primary human keratinocytes. Genome Res..

[13] Kho E.-Y., Wang H.-K., Banerjee N.S., Broker T.R., Chow L.T. (2013). HPV-18 E6 mutants reveal p53 modulation of viral DNA amplification in organotypic cultures. Proc. Natl. Acad. Sci. USA.

[14] Cimprich K.A., Cortez D. (2008). ATR: An essential regulator of genome integrity. Nat. Rev. Mol. Cell Boil..

[15] Maya-Mendoza A., Petermann E., Gillespie D.A.F., Caldecott K.W., A Jackson D. (2007). Chk1 regulates the density of active replication origins during the vertebrate S phase. EMBO J..

[16] Petermann E., Maya-Mendoza A., Zachos G., Gillespie D.A.F., Jackson D.A., Caldecott K.W. (2006). Chk1 Requirement for High Global Rates of Replication Fork Progression during Normal Vertebrate S Phase. Mol. Cell. Boil..

[17] Ward I.M., Chen J. (2001). Histone H2AX Is Phosphorylated in an ATR-dependent Manner in Response to Replicational Stress. J. Boil. Chem..

[18] Chanoux R.A., Yin B., Urtishak K.A., Asare A., Bassing C.H., Brown E.J. (2009). ATR and H2AX cooperate in maintaining genome stability under replication stress. J. Biol. Chem..

[19] Maréchal A., Zou L. (2013). DNA Damage Sensing by the ATM and ATR Kinases. Cold Spring Harb. Perspect. Boil..

[20] Anacker D.C., Gautam D., Gillespie K.A., Chappell W.H., Moody C.A. (2014). Productive Replication of Human Papillomavirus 31 Requires DNA Repair Factor Nbs. J. Virol..

[21] Banerjee N.S., Wang H.-K., Broker T.R., Chow L.T. (2011). Human Papillomavirus (HPV) E7 Induces Prolonged G2 following S Phase Reentry in Differentiated Human Keratinocytes*. J. Boil. Chem..

[22] Chappell W.H., Gautam D., Ok S.T., Johnson B.A., Anacker D.C., Moody C.A. (2016). Homologous Recombination Repair Factors Rad51 and BRCA1 Are Necessary for Productive Replication of Human Papillomavirus. J. Virol..

[23] Edwards T.G., Vidmar T.J., Koeller K., Bashkin J.K., Fisher C. (2013). DNA Damage Repair Genes Controlling Human Papillomavirus (HPV) Episome Levels under Conditions of Stability and Extreme Instability. PLoS ONE.

[24] Moody C.A., Laimins L.A. (2009). Human Papillomaviruses Activate the ATM DNA Damage Pathway for Viral Genome Amplification upon Differentiation. PLoS Pathog..

[25] Demers G.W., Foster S.A., Halbert C.L., Galloway D.A. (1994). Growth arrest by induction of p53 in DNA damaged keratinocytes is bypassed by human papillomavirus 16 E. Proc. Natl. Acad. Sci. USA.

[26] Eichten A., Westfall M., Pietenpol J.A., Munger K. (2002). Stabilization and Functional Impairment of the Tumor Suppressor p53 by the Human Papillomavirus Type 16 E7 Oncoprotein. Virology.

[27] Jian Y., Schmidt-Grimminger D.-C., Chien W.-M., Wu X., Broker T.R., Chow L.T. (1998). Post-transcriptional induction of p21cip1 protein by human papillomavirus E7 inhibits unscheduled DNA synthesis reactivated in differentiated keratinocytes. Oncogene.

[28] Kudoh A., Iwahori S., Sato Y., Nakayama S., Isomura H., Murata T., Tsurumi T. (2009). Homologous Recombinational Repair Factors Are Recruited and Loaded onto the Viral DNA Genome in Epstein-Barr Virus Replication Compartments▿. J. Virol..

[29] McKinney C.C., Hussmann K.L., McBride A.A. (2015). The Role of the DNA Damage Response throughout the Papillomavirus Life Cycle. Viruses.

[30] Smith J., Tho L.M., Xu N., Gillespie D.A. (2010). The ATM–Chk2 and ATR–Chk1 Pathways in DNA Damage Signaling and Cancer. Adv. Cancer Res..

[31] Daud A.I., Ashworth M.T., Strosberg J., Goldman J.W., Mendelson D., Springett G., Venook A.P., Loechner S., Rosen L.S., Shanahan F. (2015). Phase I Dose-Escalation Trial of Checkpoint Kinase 1 Inhibitor MK-8776 As Monotherapy and in Combination With Gemcitabine in Patients With Advanced Solid Tumors. J. Clin. Oncol..

[32] Webster J.A., Tibes R., Morris L., Blackford A.L., Litzow M., Patnaik M., Rosner G.L., Gojo I., Kinders R., Wang L. (2017). Randomized phase II trial of cytosine arabinoside with and without the CHK1 inhibitor MK-8776 in relapsed and refractory acute myeloid leukemia. Leuk. Res..

[33] Sen T., Della Corte C.M., Milutinovic S., Cardnell R.J., Diao L., Ramkumar K., Gay C.M., Stewart C.A., Fan Y., Shen L. (2019). Combination Treatment of the Oral CHK1 Inhibitor, SRA737, and Low-Dose Gemcitabine Enhances the Effect of Programmed Death Ligand 1 Blockade by Modulating the Immune Microenvironment in SCLC. J. Thorac. Oncol..

[34] Angius G., Tomao S., Stati V., Vici P., Bianco V., Tomao F. (2019). Prexasertib, a checkpoint kinase inhibitor: from preclinical data to clinical development. Cancer Chemother. Pharmacol..

[35] Guzi T.J., Paruch K., Dwyer M.P., Labroli M., Shanahan F., Davis N., Taricani L., Wiswell D., Seghezzi W., Penaflor E. (2011). Targeting the Replication Checkpoint Using SCH 900776, a Potent and Functionally Selective CHK1 Inhibitor Identified via High Content Screening. Mol. Cancer Ther..

[36] Albigès L., Goubar A., Scott V., Vicier C., Lefebvre C., Alsafadi S., Commo F., Saghatchian M., Lazar V., Dessen P. (2014). Chk1 as a new therapeutic target in triple-negative breast cancer. Breast.

[37] Lei H., Jin J., Liu M., Li X., Luo H., Yang L., Xu H., Wu Y. (2018). Chk1 inhibitors overcome imatinib resistance in chronic myeloid leukemia cells. Leuk. Res..

[38] Zhou Z.-R., Yang Z.-Z., Wang S.-J., Zhang L., Luo J.-R., Feng Y., Yu X.-L., Chen X.-X., Guo X.-M. (2017). The Chk1 inhibitor MK-8776 increases the radiosensitivity of human triple-negative breast cancer by inhibiting autophagy. Acta Pharmacol. Sin..

[39] Banerjee N.S., Wang H.-K., Beadle J.R., Hostetler K.Y., Chow L.T. (2017). Evaluation of ODE-Bn-PMEG, an acyclic nucleoside phosphonate prodrug, as an antiviral against productive HPV infection in 3D organotypic epithelial cultures. Antivir. Res..

[40] Engelke C.G., Parsels L.A., Qian Y., Zhang Q., Karnak D., Robertson J.R., Tanska D.M., Wei D., Davis M.A., Parsels J.D. (2013). Sensitization of pancreatic cancer to chemoradiation by the Chk1 inhibitor MK. Clin. Cancer Res..

[41] Leung-Pineda V., Ryan C.E., Piwnica-Worms H. (2006). Phosphorylation of Chk1 by ATR Is Antagonized by a Chk1-Regulated Protein Phosphatase 2A Circuit†. Mol. Cell. Boil..

[42] Bakkenist C.J., Kastan M.B. (2003). DNA damage activates ATM through intermolecular autophosphorylation and dimer dissociation. Nature.

[43] Mukherjee B., Kessinger C., Kobayashi J., Chen B.P., Chen D.J., Chatterjee A., Burma S. (2006). DNA-PK phosphorylates histone H2AX during apoptotic DNA fragmentation in mammalian cells. DNA Repair.

[44] Banerjee N.S., Moore D.W., Broker T.R., Chow L.T. (2018). Vorinostat, a pan-HDAC inhibitor, abrogates productive HPV-18 DNA amplification. Proc. Natl. Acad. Sci. USA.

[45] Banerjee N.S., Moore D.W., Wang H.-K., Broker T.R., Chow L.T. (2019). NVN1000, a novel nitric oxide-releasing compound, inhibits HPV-18 virus production by interfering with E6 and E7 oncoprotein functions. Antivir. Res..

[46] Cheng S., Schmidt-Grimminger D.C., Murant T., Broker T.R., Chow L.T. (1995). Differentiation-dependent up-regulation of the human papillomavirus E7 gene reactivates cellular DNA replication in suprabasal differentiated keratinocytes. Genes Dev..

[47] Suzuki M., Yamamori T., Bo T., Sakai Y., Inanami O. (2017). MK-8776, a novel Chk1 inhibitor, exhibits an improved radiosensitizing effect compared to UCN-01 by exacerbating radiation-induced aberrant mitosis. Transl. Oncol..

[48] Sowd G.A., Li N.Y., Fanning E. (2013). ATM and ATR Activities Maintain Replication Fork Integrity during SV40 Chromatin Replication. PLoS Pathog..

[49] Hardouin N., Nagy A. (2000). Gene-trap-based target site for cre-mediated transgenic insertion. Genesis.

[50] Banerjee N.S., Chow L.T., Broker T.R. (2005). Retrovirus-mediated gene transfer to analyze HPV gene regulation and protein functions in organotypic “raft” cultures. Methods Mol. Med..

